# Mechanistic Review of Topical Therapies Targeting Dermal Fibroblasts in Skin Aging and Rejuvenation

**DOI:** 10.1111/jocd.70987

**Published:** 2026-06-17

**Authors:** Seyedeh Melika Akaberi, Mohammad Esfandiyari, Hamid Reza Ahmadi Ashtiani

**Affiliations:** ^1^ Department of Pathobiology, Cummings School of Veterinary Medicine Tufts University North Grafton Massachusetts USA; ^2^ Department of Biochemistry, TeMS.C. Islamic Azad University Tehran Iran; ^3^ Cosmetic, Hygienic and Detergent Sciences and Technology Research Center, TeMS.C. Islamic Azad University Tehran Iran

**Keywords:** cosmetics, dermal fibroblasts, fibroblast signaling, skin aging, topical treatments

## Abstract

**Background:**

Skin aging appears in multiple forms, including wrinkles, sagging, scarring, atrophy, and photoaging. Although each phenotype has distinct biological features, all are rooted in dermal fibroblast dysfunction. This review examines the molecular pathways underlying these conditions and evaluates topical interventions designed to restore skin integrity.

**Aims:**

To assess how dermal fibroblast dysfunction contributes to different skin aging phenotypes and to evaluate topical therapies targeting the underlying molecular mechanisms.

**Methods:**

A narrative review of the literature was conducted focusing on fibroblast‐related molecular pathways and topical interventions used for skin aging and rejuvenation.

**Results:**

UVB radiation contributes to wrinkle formation by activating collagen‐degrading enzymes in the upper dermis, whereas sagging results from UVA‐induced damage to deeper connective tissues and altered fat distribution. Scarring is characterized by excessive fibroblast activation and extracellular matrix overproduction, while atrophy develops through fibroblast senescence and loss of structural support. Photoaging involves oxidative stress and elastin disorganization caused by chronic ultraviolet exposure. Topical agents including retinol, niacinamide, caffeine, asiaticoside, and related compounds stimulate collagen synthesis, reduce matrix degradation, and protect fibroblasts from oxidative damage. Unlike previous reviews that examine skin aging as a single process, this review compares distinct aging phenotypes through the common framework of fibroblast dysfunction and their associated molecular targets.

**Conclusions:**

Dermal fibroblast dysfunction is a central mechanism underlying multiple forms of skin aging. Targeting fibroblast‐related pathways with topical therapies may address the biological causes of wrinkles, sagging, scarring, atrophy, and photoaging, supporting more personalized and effective skin rejuvenation strategies.

## 1 Introduction

Skin aging results from internal and external factors and appears as wrinkles, sagging, scarring, atrophy, and photoaging. These visible signs affect not only appearance but also skin structure and function [1]. The main cause is fibroblast dysfunction, cells that maintain the extracellular matrix (ECM), which worsen over time due to oxidative stress, senescence, and signaling disruptions [2].

Intrinsic aging, which is regulated by hormonal and genetic changes, progressively reduces fibroblast activity, which in turn decreases the production of collagen and elastin. This leads to weaker, thinner, and less elastic skin. These effects are intensified by extrinsic factors, especially extended exposure to ultraviolet (UV) radiation. UV light speeds up the breakdown of collagen and elastin by producing reactive oxygen species (ROS), disrupting fibroblast signaling pathways, and encouraging matrix metalloproteinases (MMPs) to break down ECM [3].

Different mechanisms of fibroblast dysfunction are involved in different aging phenotypes. UVA rays, for instance, impact deeper connective tissues and subcutaneous fat, resulting in sagging, whereas UVB rays contribute to the breakdown of superficial collagen, causing wrinkles. Skin atrophy is caused by aging cells with diminished fibroblast function, while hyperactive fibroblasts and dysregulated transforming growth factor‐β (TGF‐β) signaling cause scarring. Long‐term exposure to sunlight causes photoaging, which is typified by oxidative damage and extensive disruption of the ECM [2, 3, 4, 5, 6, 7].

Despite growing understanding, the lack of direct comparisons between molecular mechanisms of aging phenotypes limits development of targeted therapies [8]. Topical agents like retinol, caffeine, and silicone gels interact with fibroblast pathways [9, 10, 11], but are often used broadly, without phenotype‐specific application.

This review aims to:

1. Compare five distinct signs of skin aging and their underlying fibroblast‐related triggers;
2. Map molecular differences, including ROS, MMP, and TGF‐β activity;
3. Evaluate treatments based on how precisely they target fibroblast dysfunction.

By adopting a targeted, mechanism‐driven approach, this review emphasizes tailored therapies that address the biological causes of aging rather than superficial symptoms, paving the way for personalized skincare solutions.

## 2 Materials and Methods

This review focused on articles published predominantly between 2019 and 2024, reflecting recent advancements in the field. However, older studies were included when essential data were unavailable in newer literature. Advanced searches were conducted on databases such as PubMed and Google Scholar, and also AI‐powered tools like Scispace, with keywords including “Skin Aging, Cosmetics, Topical Treatments, Fibroblast Signaling, and Dermal Fibroblasts” to ensure a comprehensive integration of key mechanisms and therapeutic strategies.

## 3 Dermal Fibroblasts and Wrinkle Formation

Wrinkles appear as visible lines on sun‐exposed areas like the face and neck, due to a combination of internal aging and external stressors that alters dermal structure [1, 12]. They are primarily caused by progressive ECM degradation and mechanical instability at the skin's surface [3, 13]. This section highlights the specific contribution of dermal fibroblast dysfunction to wrinkle development.

### 3.1 Molecular Mechanisms of Wrinkle Formation

Dermal fibroblasts play a crucial role in maintaining ECM by synthesizing key components such as collagen types I and III, elastin, glycosaminoglycans, and proteoglycans. These biomolecules preserve tensile strength, elasticity, and hydration of skin [14, 15]. In wrinkles, fibroblast function is impaired by intrinsic aging and by UVB exposure, which stimulates oxidative stress and ECM breakdown [1, 12]. Histologically, wrinkles are a flattening of the dermal‐epidermal junction, thinning of the reticular dermis, and fragmented elastic fibers [3, 12].

UVB radiation stimulates mitogen‐activated protein kinase (MAPK) pathways (ERK, p38, JNK), resulting in Activator Protein‐1 (AP‐1) and NF‐κB activation and overexpression of MMPs (MMP‐1, ‐2, ‐3, and ‐9) [16, 17]. These enzymes degrade ECM proteins, reducing cutaneous support. Fibroblasts generate neprilysin, an enzyme that promotes ECM breakdown through keratinocyte signals via interleukin‐1α (IL‐1α) [18, 19]. Fibroblasts in wrinkle development can activate MMP‐1 via plasmin without the usual wound‐healing signals [20].

Wrinkle development is linked to disturbed TGF‐β signaling, which inhibits COL1A1 and COL3A1 production. As the ECM degrades, fibroblasts lose attachment cues, causing greater dysfunction, increased ROS and MMP production, and further damage [20, 21].

### 3.2 Topical Treatments for Wrinkle Formation

Topical therapy for wrinkle‐associated fibroblast dysfunction attempts to restore collagen production, limit MMP activity, and reduce oxidative stress via non‐invasive techniques.

- *Retinol* promotes TGF‐β signaling, increases collagen production (COL1A1, COL3A1), and inhibits AP‐1 to decrease MMP‐1 and reduce ROS damage [10, 22].
- *Niacinamide* reduces ROS, inhibits AP‐1 and NF‐κB, and increases procollagen levels, improving skin elasticity [23].
- *Galangin* protects against UVB‐induced ROS, supports TGF‐β signaling, and reduces MMP‐1 expression, preserving collagen integrity [24].
- *Hyaluronic Acid (HA)* promotes fibroblast proliferation, suppressing MMP‐1 and enhancing ECM components via TGF‐β‐mediated pathways and connective tissue growth factor (CTGF) upregulation [25].
- *(PDRN)* activates TGF‐β signaling, increasing collagen synthesis while decreasing MMP‐mediated ECM degradation [26].
- *XEP‐716* mimics TGF‐β, enhancing collagen, elastin, and HA production while suppressing MMPs [27].

These treatments, which target wrinkle‐specific fibroblast dysfunction, help restore skin structure and reduce visible aging signs effectively. A summary of the key pathways and fibroblast dysfunctions involved in wrinkle formation is presented in Table 1.

**TABLE 1:** Key pathways and fibroblast dysfunctions driving wrinkle formation.

Aspect	Key points	Effects	References
Causes of wrinkle formation	UVB + intrinsic factors cause ECM breakdown	↑ ROS ↑ MMP‐1 ↓ COL1A1 ↓ elastin	[1, 3, 12, 17]
Role of fibroblasts	ECM synthesis declines, elastase ↑	↓ Collagen/elastin ↑ MMP‐1/neprilysin	[17, 18, 19]
Signaling pathways	ROS → MAPK → AP‐1/NF‐κB → MMPs ↑	↑ MMP‐1 ↑ MMP‐3 ↑ MMP‐9	[17, 21, 28]

## 4 Dermal Fibroblasts and Skin Sagging

Skin sagging, the drooping of facial features like cheeks and jawline, is a key sign of aging caused by structural damage in deeper subcutaneous layers [29, 30]. Unlike wrinkles, from ECM surface damage, sagging results from multiple factors: RC weakness, increased fat, and reduced muscle activity in expressions [4, 29]. This section examines dermal fibroblasts' role in sagging formation.

### 4.1 Molecular Mechanisms of Skin Sagging

Fibroblasts are essential for skin structure as they produce collagen and elastin, providing strength and elasticity and supporting the retinacula cutis, a collagen network linking the dermis to fat layers underneath [7].

In the context of sagging, UVA exposure damages deeper dermis layers, elastic fibers, and causes visible signs [31]. UVA‐generated ROS accelerate DNA damage in fibroblasts, activating the senescence‐associated secretory phenotype (SASP), causing inflammation and ECM breakdown [31, 32]. UVA also stimulates the MAPK/AP‐1 and mechanistic target of rapamycin (mTOR) pathways, increasing MMP‐1 expression and disrupting IGF‐1 and TGF‐β signaling, further reducing collagen production [7, 31, 32].

Sagging results from deep reticular collagen breakdown and fat displacement beneath the skin. Unlike wrinkles, caused by UVB‐induced collagen damage at the dermal‐epidermal junction [5, 13], sagging reflects deeper structural loss and fat imbalance. Addressing these mechanisms highlights the need for treatments targeting subcutaneous tissue integrity and fat redistribution [4, 29].

### 4.2 Topical Treatments for Skin Sagging

Treatments for sagging skin aim to restore fibroblast function by stimulating collagen production, balancing fat tissue, reducing oxidative stress, and inhibiting MMPs that damage the skin's structure.

- *Caffeine* stimulates lipolysis by inhibiting phosphodiesterase, reducing subcutaneous fat and enhancing skin firmness. The antioxidant properties provide protection against ROS damage [9, 33].
-
*Centella Asiatica* (Asiaticoside) promotes fibroblast collagen production through PI3K/AKT and NF‐κB pathways, suppresses MMP‐1 and MMP‐2, and reduces SASP‐induced inflammation, thereby maintaining the ECM [34, 35, 36].
- *Palmitoyl Pentapeptide‐4* activates TGF‐β/SMAD signaling, increasing collagen and elastin synthesis while stabilizing the ECM by inhibiting MMP‐1 and MMP‐3. Clinical studies reveal its efficacy in reducing periorbital sagging [37, 38, 39].
- *Resveratrol* enhances autophagy and inhibits ROS‐induced fibroblast senescence through the activation of AMPK. It stimulates collagen synthesis through estrogen receptor signaling and downregulates MMPs via MAPK pathways, enhancing skin elasticity [40, 41, 42].

These interventions target fibroblast dysfunction to effectively restore skin firmness and structural integrity. The major molecular pathways and fibroblast‐related mechanisms involved in skin sagging are summarized in Table 2.

**TABLE 2:** Key pathways and fibroblast dysfunctions driving skin sagging.

Aspect	Key points	Effects	References
Causes of sagging	UVA exposure and intrinsic aging impair fibroblast function and weaken retinacula cutis (RC) anchoring	↑ ROS ↑ MMP‐1 ↓ COL1A1 ↓ Elastin ↓ IGF‐1 ↑ mTOR	[4, 7, 29, 32, 40]
Role of fibroblasts	Responsible for deep dermal collagen and RC structure; senescence triggered by UVA and SASP	↓ ECM synthesis ↑ MMPs ↓ TGF‐β signaling ↓ IGF‐1 signaling ↓ dermal tension	[7, 29, 32]
Signaling pathways	ROS → MAPK/AP‐1 activation; SASP inflammation; ↓ TGF‐β and IGF‐1; ↑ mTOR in fibroblasts	ECM breakdown fibroblast aging RC disintegration chronic inflammation	[29, 31, 32, 43]

## 5 Dermal Fibroblasts and Scar Hypertrophy

Scar tissue hypertrophy, including hypertrophic scars and keloids, is characterized by elevated, fibrous lesions due to dysregulated wound healing and excessive ECM accumulation [6, 44]. Prolonged myofibroblast activation and prolonged inflammatory responses characterize hypertrophic scars [8, 45]. This section investigates the function of dermal fibroblasts in scar hypertrophy.

### 5.1 Molecular Mechanisms of Scar Hypertrophy

Dermal fibroblasts are essential to wound healing, differentiating into myofibroblasts that produce collagen types I and III, fibronectin, and other ECM components [46, 47]. In hypertrophic scars and keloids, dysregulated TGF‐β signaling, primarily through TGF‐β1 and its receptors, maintains myofibroblast activity, driving excessive collagen synthesis and fibrotic ECM accumulation [8, 48]. CTGF, upregulated by TGF‐β, further facilitates these mechanisms [48].

Inflammation plays an essential role in scar hypertrophy. Lipopolysaccharide triggers fibroblast‐mediated TLR4/MyD88 signaling, leading to IL‐6 and TNF‐α secretion, which sustain myofibroblast activity [49]. Paracrine interactions between keratinocytes and fibroblasts enhance fibrosis via IL‐1α and TGF‐β [45]. Scar hypertrophy is characterized by mechanical stress and low oxygen levels, which increase α‐smooth muscle actin (α‐SMA) levels in myofibroblasts, thereby enhancing contractility and the stiffness of ECM [50, 51]. Non‐coding RNAs, particularly miR‐21, regulate fibroblast activity, with miR‐21 driving collagen overproduction in keloids [52].

### 5.2 Topical Treatments for Scar Hypertrophy

Topical treatments for scar hypertrophy focus on regulating myofibroblast activity, ECM formation, and inflammation.

- *Silicone‐based gels* (e.g., dimethylpolysiloxane) create a protective barrier that preserves moisture, regulates fibroblast activity, and downregulates TGF‐β1, reducing type I and III collagen production [53, 54, 55]. Silicone also inhibits myofibroblast differentiation, suppresses IL‐6 and TNF‐α, and disrupts paracrine signaling between epidermal cells and fibroblasts, reducing inflammation. Its application, alone or combined with allantoin, dexpanthenol, or heparin, decreases scar elevation within 28 days in models, and clinical trials indicate improved elasticity and reduced scar thickness following 8–12 weeks [53, 55].

Through modulating fibroblast activity and ECM production, silicone remains one of the most effective topical options for managing hypertrophic scars and improving overall scar appearance. Table 3 summarizes the principal signaling pathways and fibroblast dysfunctions associated with scar hypertrophy.

**TABLE 3:** Key pathways and fibroblast dysfunctions driving scar hypertrophy.

Aspect	Key points	Effects	References
Causes of hypertrophic scarring	TGF‐β1 overactivation promotes persistent myofibroblast activity and collagen overproduction	↑ TGF‐β1 ↑ CTGF ↑ COL1A1/COL3A1	[6, 8, 48]
Role of fibroblasts	Differentiation into myofibroblasts with enhanced contractility and fibrotic signaling	↑ α‐SMA ↑ ECM stiffness ↑ fibrosis	[44, 47, 49, 50]
Signaling pathways	LPS → TLR4/MyD88 → IL‐6, TNF‐α; keratinocyte‐fibroblast crosstalk via IL‐1α/TGF‐β	Sustained inflammation myofibroblast persistence ECM remodeling	[45, 49, 50, 51, 52]

## 6 Dermal Fibroblasts and Skin Atrophy

Skin atrophy results from reduced fibroblast function and ECM degradation, defined by dermal thinning, reduced structural stiffness, and elasticity loss [2, 3]. This section highlights the essential role of fibroblasts in atrophy and investigates its mechanistic foundations.

### 6.1 Molecular Mechanisms of Skin Atrophy

In skin atrophy, senescent fibroblasts display a SASP, secreting pro‐inflammatory cytokines (IL‐6, IL‐8), MMP‐1 and MMP‐3, which damage collagen and elastin, resulting in dermal thinning [3]. Also, external factors, primarily associated with oxidative stress from ROS, induce fibroblast senescence through mitochondrial dysfunction and DNA damage [56].

Inhibited TGF‐β signaling due to decreased receptor expression restricts collagen production, while lower fibroblast growth factors (FGFs) limit cell proliferation and ECM formation [3, 57]. Inflammation triggered by SASP and loss of mechanical tension further accelerates ECM breakdown via weakened integrin signaling [58]. Interactions between epidermal cells and fibroblasts, mediated by keratinocyte‐derived IL‐6, increase MMP production, resulting in accelerating skin thinning [3].

### 6.2 Topical Treatments for Skin Atrophy

Topical cosmetics counteract skin atrophy by regulating fibroblast senescence, oxidative stress, and ECM degradation [59].

- *Calcipotriol*, a vitamin D analog that combats corticosteroid‐induced ECM loss by enhancing fibroblast activity, promoting TGF‐β signaling, and increasing collagen type I production. It inhibits MMP‐1, MMP‐3, and SASP‐related cytokines to reduce inflammation‐driven ECM degradation. In vitro studies confirm ECM restoration within 2 weeks of treatment [3, 60].
- *Polycomponent formulations*, comprising peptides, antioxidants, and growth factors, rejuvenate aged fibroblasts by mitigating oxidative stress and inflammatory pathways. They upregulate FGF and vascular endothelial growth factor (VEGF) signaling, boost fibroblast activity, and increase collagen type I synthesis (30% in 7 days, in vitro) [59]. Antioxidants like vitamin C neutralize ROS, and peptides mimic TGF‐β signaling to rebuild the ECM [59, 61].

These interventions directly address core mechanisms of atrophy, supporting dermal regeneration and function. A concise overview of the molecular mechanisms and fibroblast dysfunctions contributing to skin atrophy is provided in Table 4.

**TABLE 4:** Key pathways and fibroblast dysfunctions driving skin atrophy.

Aspect	Key points	Effects	References
Causes of atrophy	Intrinsic aging and oxidative stress induce fibroblast senescence and ECM loss	↑ ROS ↑ SASP cytokines (IL‐6, IL‐8) ↓ TGF‐β ↓ FGFs	[2, 3, 56, 57, 62]
Role of fibroblasts	Senescent fibroblasts secrete MMPs and inflammatory mediators; ECM synthesis declines	↑ MMP‐1/MMP‐3 ↓ COL1A1 ↓ proliferation ↑ inflammation	[3, 58, 62]
Signaling pathways	DNA damage → SASP → MMPs + cytokines; ↓ TGF‐βR and integrin signaling	ECM degradation Thinning of dermis Impaired fibroblast–ECM crosstalk	[3, 57, 58]

## 7 Dermal Fibroblasts and Photoaging

Photoaging results from prolonged exposure to UVA/UVB, presenting as deep wrinkles, hyperpigmentation, and solar elastosis [4, 63]. It manifests as a complex interplay of oxidative stress and elastin fiber disorganization, driving significant molecular disruptions in dermal fibroblasts [64, 65].

### 7.1 Molecular Mechanisms of Photoaging

Chronic UVA/UVB radiation generates ROS, damaging fibroblast DNA, proteins, and mitochondria [16, 66]. Oxidative stress induced by ROS activates the MAPK/AP‐1 pathway, leading to the upregulation of MMP‐1, MMP‐3, and MMP‐9, which drive ECM degradation [65]. Also, UVB disrupts collagen production by suppressing TGF‐β signaling through downregulating its receptor (TGF‐βRII) expression [63].

UVA radiation produces 4‐hydroxynonenal (4‐HNE), a lipid peroxidation byproduct, accelerating fibroblast senescence via SASP secretion, which promotes chronic inflammation [67]. Solar elastosis arises from elastin fiber degradation, driven by fibrillin and versican damage from elastase activity [64, 67]. The impaired nuclear factor erythroid 2‐related factor 2 (Nrf2)/heme oxygenase‐1 (HO‐1) pathway diminishes fibroblast resistance to ROS [68, 69]. Unlike UVB‐initiated wrinkle formation via AP‐1 signaling, UVA‐driven photoaging involves MAPK activation, mitochondrial dysfunction, post‐translational fibroblast modifications, and elastin degradation [69].

### 7.2 Topical Treatments for Photoaging

Topical treatments for photoaging mitigate UVA/UVB‐induced ROS, ECM degradation, and elastin disorganization [4, 70].

- *Puerarin*, a flavonoid that protects fibroblasts from UVA‐induced damage by activating Nrf2/HO‐1 signaling, neutralizing ROS, and suppressing MAPK/ERK pathways, reducing MMP‐1 levels and enhancing collagen type I via TGF‐β signaling. In vitro, it decreased fibroblast senescence markers by 25% within 48 h [71].
- *3,5‐dicaffeoyl‐epi‐quinic acid*, a polyphenol that reduces photoaging by scavenging ROS, inhibiting MMP‐1/9 through MAPK and JNK pathways, stabilizing fibrillin‐1, reducing elastase activity, and increasing elastin deposition by 30% after 72 h [72].
- *SA1‐III peptide*, mimics TGF‐β signaling to boost collagen and pro‐elastin production, suppress AP‐1, and reduce MMP‐1/3. Clinical trials showed a 20% increase in skin elasticity after 8 weeks, supported by heat shock protein‐47 (HSP‐47) induction [73].

These active ingredients enhance fibroblast performance, preserving skin elasticity and structure. The major fibroblast‐related pathways involved in photoaging are summarized in Table 5.

**TABLE 5:** Key pathways and fibroblast dysfunctions driving photoaging.

Aspect	Key points	Effects	References
Causes of photoaging	Chronic UVA/UVB exposure induces oxidative stress and ECM disorganization	↑ ROS ↑ 4‐HNE ↑ MMP‐1/‐3/‐9 ↓ TGF‐βRII ↓ Nrf2/HO‐1	[4, 16, 63, 65, 67, 68, 71]
Role of fibroblasts	Fibroblasts undergo oxidative damage, senescence (SASP), and impaired elastogenesis	↑ IL‐6/IL‐8 ↓ COL1A1 Disordered elastin ↑ elastase	[16, 64, 66, 67]
Signaling pathways	UVA/UVB → ROS → MAPK/AP‐1 → MMPs; ↓ TGF‐β/Smad; ↓ Nrf2 → antioxidant collapse	ECM degradation Solar elastosis Impaired repair capacity	[63, 65, 69, 71]

Table 6 provides a comparative overview of topical therapeutics targeting fibroblast dysfunction across different skin aging phenotypes.

**TABLE 6:** Topical therapeutics targeting fibroblast dysfunction across skin aging phenotypes.

Aging phenotype	Ingredients	Mechanisms	Effects on fibroblasts/ECM	Clinical benefits	References
Wrinkle	Retinol	↑ TGF‐β signaling ↓ AP‐1 ↑ SOD	↑ Collagen ↓ MMP‐1 ↓ ROS	Improves collagen synthesis	[10, 22]
	Niacinamide	↑ PPARGC1B ↓ ROS inhibits AP‐1/NF‐κB	↓ MMP‐1/MMP‐9 ↑ collagen synthesis	Reduces wrinkle severity	[23]
	Galangin	ROS scavenger ↑ TGF‐β/SMAD	↓ MMP‐1 protects mitochondria	Prevents UVB‐induced damage	[24]
	Hyaluronic acid	↑ Fibroblast proliferation ↑ CTGF	↓ MMP‐1 supports ECM hydration	Enhances skin hydration	[25]
	PDRN	Activates A2A receptor → ↑ TGF‐β	↓ NF‐κB ↓ MMP‐1/MMP‐9	Improves skin elasticity	[26]
	XEP‐716	TGF‐β mimicry, ↑ COL1A1/ELN/HAS2	↓ MMP‐1	Increases skin firmness	[27]
Skin sagging	Caffeine	Inhibits phosphodiesterase → ↑ cAMP → lipolysis; mild antioxidant effect	↓ Subcutaneous fat Indirect support for RC	Reduces facial sagging Improves dermal firmness	[9, 33]
	Asiaticoside	Modulates PI3K/AKT and NF‐κB pathways; inhibits MMP‐1/‐2; antioxidant/anti‐inflammatory	↑ Collagen and elastin synthesis ↓ SASP; Protects RC structure	Enhances skin elasticity Shown to firm skin clinically	[34, 35, 36]
	Palmitoyl pentapeptide‐4	Activates TGF‐β/SMAD signaling; inhibits MMP‐1/‐3	↑ Type I collagen and elastin; restores RC architecture	Reduces periorbital sagging Improves firmness	[37, 38, 39]
	Resveratrol	Activates AMPK, SIRT1, and Nrf2; inhibits MAPK and COX‐2; induces autophagy	↓ Fibroblast senescence ↑ collagen synthesis ↓ SASP‐related inflammation	Boosts skin elasticity Protects deep ECM	[40, 41, 42, 74]
Scar hypertrophy	Silicone‐based gels	Forms occlusive barrier; maintains hydration; inhibits TGF‐β1 and inflammatory cytokines	↓ TGF‐β1 ↓ IL‐6/TNF‐α ↓ myofibroblast differentiation ↓ COL I/III	Reduces scar thickness Improves elasticity	[53, 54, 55]
Skin atrophy	Calcipotriol	Activates TGF‐β signaling; inhibits SASP‐related cytokines; counteracts corticosteroid‐induced atrophy	↑ COL1A1 ↓ MMP‐1/MMP‐3 ↓ IL‐6/IL‐8	Prevents ECM loss Restores dermal thickness	[60]
	Polycomponent formulations (peptides + antioxidants + growth factors)	↑ FGF/VEGF expression; mimics TGF‐ β signaling; reduces oxidative and inflammatory markers	↑ Fibroblast proliferation ↑ collagen synthesis ↓ ROS	Enhances skin resilience Restores elasticity and ECM structure	[59, 61]
Photoaging	Puerarin	Activates Nrf2/HO‐1; inhibits MAPK/ERK; activates TGF‐ β signaling	↓ ROS ↓ MMP‐1 ↑ COL1A1	Reduces fibroblast senescence Restores antioxidant defense	[71]
	3,5‐dicaffeoyl‐epi‐quinic acid	Scavenges ROS; inhibits p38 MAPK/JNK; stabilizes fibrillin‐1	↓ MMP‐1/‐9 ↓ Elastase Preserves elastin network	Prevents solar elastosis Increases elastin deposition	[72]
	SA1‐III peptide	Mimics TGF‐ β; ↑ collagen & pro‐elastin synthesis; ↓ AP‐1 and MMPs	↑ HSP‐47 ↑ COL1A1 ↓ MMP‐1/‐3	Improves elasticity and ECM structure	[73]

## 8 Discussion

Fibroblast dysfunction is the foundation of various aging phenotypes, emphasizing their shared pathological mechanisms. Wrinkles result from UVB‐induced ECM degradation in the superficial dermis through MMP activation and reduced TGF‐β signaling [3, 13, 16]. Sagging is driven by UVA‐induced ROS and SASP activation, which disrupt IGF‐1 and TGF‐β pathways, causing deeper tissue alterations and fat redistribution [29, 31]. Hyperactive fibroblasts with dysregulated TGF‐β1 signaling contribute to scarring through excessive collagen deposition [6, 46]. Atrophy reflects fibroblast senescence, marked by reduced ECM production, increased oxidative stress, and elevated inflammatory cytokines [2, 3, 60]. UV‐driven ROS damage combined with elastin fiber disorganization defines photoaging, impairing both collagen and elastin function via MAPK and AP‐1 pathways [63, 64, 71].

Fibroblast‐targeted therapies are fundamental in addressing skin aging. Strategies involve reducing ROS to mitigate wrinkles [22] and sagging [29], suppressing fibroblast overactivity in scars [54], and restoring functionality to senescent fibroblasts in atrophic skin [3, 60]. Topical applications of asiaticoside, retinol, and peptides stimulate fibroblast activity, driving ECM production and regulating inflammation [22, 73, 75].

Future advancements in regenerative skincare involve fibroblast reprogramming via stem‐cell‐derived exosomes, epigenetic modulators, gene editing, and nano‐delivery systems, addressing localized regeneration and bioavailability. Direct fibroblast applications and their exosomes also represent promising therapeutic tools, though challenges like high costs and limited clinical validation for long‐term safety persist.

## 9 Conclusion

This study emphasizes the significance of fibroblast dysfunction in the onset of wrinkles, sagging, scarring, and atrophy. Skin aging symptoms result from a complicated interaction between intrinsic aging and external stresses, impairing fibroblast‐mediated ECM maintenance, signaling, and repair.

Current topical therapies exhibit potential in enhancing collagen synthesis, providing antioxidant advantages, and suppressing MMPs. While innovative approaches such as gene‐editing technologies, stem‐cell exosomes, and epigenetic therapies present promising opportunities for attaining precise and individualized regeneration of skin. Topical therapy employing autologous fibroblasts effectively utilizes the cell's regenerative potential to repair skin structure and function. The fibroblast is pivotal to skin rejuvenation, as it regulates ECM formation and cellular tissue regeneration.

In summary, fibroblast‐based strategies are essential to advance personalized and effective skincare treatments.

## Conflicts of Interest

The authors declare no conflicts of interest.

## Data Availability

The authors have nothing to report.
